# Towards healthy urbanism: inclusive, equitable and sustainable (THRIVES) − an urban design and planning framework from theory to praxis

**DOI:** 10.1080/23748834.2020.1769527

**Published:** 2020-06-26

**Authors:** Helen Pineo

**Affiliations:** Institute for Environmental Design and Engineering, Bartlett School of Environment, Energy and Resources, University College London, London, UK

**Keywords:** Urban, health and wellbeing, design, planning

## Abstract

The globally distributed health impacts of environmental degradation and widening population inequalities require a fundamental shift in understandings of healthy urbanism − including policies and decisions that shape neighbourhood and building design. The built environment tends to disadvantage or exclude women, children, the elderly, disabled, poor and other groups, starting from design and planning stages through to occupation, and this results in avoidable health impacts. Although these concepts are not new, they are rapidly emerging as built environment research and practice priorities without clear understanding of the interconnected aims of healthy environments that are sustainable, equitable and inclusive. This article promotes a new framework − Towards Healthy uRbanism: InclusiVe Equitable Sustainable (THRIVES) − that extends previous conceptualisations and reorients focus towards the existential threat of environmental breakdown and the social injustice created through inequitable and exclusive urban governance and design processes and outcomes. The Framework was developed through synthesising knowledge from research and practice, and by testing this new conceptualisation in a participatory workshop. Ongoing research is exploring implementation of the Framework in practice. If widely adopted, this Framework may contribute towards achieving the goals of sustainable development through a focus on increasing human health and wellbeing in urban development.

## Introduction

The agendas of international bodies such as the World Health Organization (WHO) and United Nations (UN) have recently converged with property development and urban planning professionals over the topic of healthy urbanism. This new healthy building and planning agenda is evident in the proliferation of guidance (e.g. World Green Building Council 2014, 2016, Urban Land Institute 2015, UN-Habitat 2018, Pineo and Rydin 2018, WHO 2018b). The achievement of both health and sustainability goals through urban development has been advocated through the parallel activities of the WHO Healthy Cities and sustainable building movements (Hancock and Duhl 1986, Reed *et al*. 2009, Rydin 2010, Hancock 2011), and historically these agendas have not intersected substantively. However, the global trends of rapid urbanisation, resource and biodiversity loss, climate change, widening inequalities, ageing populations and the rising burden of non-communicable diseases (Gatzweiler *et al*. 2017) are part of the context that has brought these aligned, yet predominately separate, fields of research and practice together. The other part of this alignment relates to new perceptions among urban development professionals about health and wellbeing. Urban planners’ increased interest in health and wellbeing (Pfeiffer and Cloutier 2016) is now met with a receptive audience among some developers and landowners who see the potential for added value or market differentiation (Chang 2018). Although economic viability remains a challenge for integrating healthy design measures and construction materials into many projects (Carmichael *et al*. 2019), the emergence of new standards, such as WELL and Fitwel, indicates a new way for healthy development to be assessed and valued (Pineo and Rydin 2018). The healthy building and planning agenda is thus shaped by many voices with diverse perspectives on how built environment professionals should be part of health promotion and protection. This article introduces a new way of conceptualising healthy urbanism that responds to perceived gaps in the existing professional knowledge base and guidance for urban development. The author argues that the concept of healthy urban development needs to be reframed to encompass the connected lenses of sustainability, equity and inclusion and the consideration of health impacts at multiple spatial and temporal dimensions.

In the context of deregulation and increased reliance on market-led development, both public and private sector actors need to have a shared understanding of the importance of healthy environments, yet this is not currently the case. A Design Council (2018) survey of 601 British planners and urban designers found that 82% of respondents’ perceived differences between their own view of healthy place-making and that of developers. Respondents felt that market pressures and insufficient funding were significant barriers, but also that healthy development was not ‘seen as the “norm”’ (p. 7). The survey highlighted a knowledge gap in how healthy place-making is conceptualised by professionals, who placed greater priority on increasing physical activity than issues such as homes for people from different backgrounds, compact mixed-use communities, indoor environments and job creation. This survey reinforces previous international research findings (see Carmichael *et al*. 2012) that gaps in knowledge and conceptual understanding are key challenges for integrating health into urban development.

Furthermore, not all built environment professionals accept responsibility for safeguarding health and sustainability, or improving inequalities, through building and urban design. In his editorial in *The Lancet*, Richard Horton (2012) described being defeated by a panel of architects who claimed ‘Architecture cannot cure the world’s ills … There is no moral duty on architects to incorporate cures into their work’ (p. 94). It is unclear how widespread these views were among built environment professionals at that time, but some disagreed (Pineo 2012). Similarly, architects’ relatively late awakening to the climate crisis has been criticised (Murray 2019). There likely remain many built environment practitioners who do not consider health and sustainability objectives to be part of their work (Pilkington *et al*. 2013, Marsh *et al*. 2020b). Unlike public health professionals who have greater consensus over their foundational values for promoting population health (Lee and Zarowsky 2015), the core purpose of built environment professions varies internationally, among individuals and across the professions. Yet the need for action is clear and increasingly heard, if not fully understood, among those working in planning, property development and regeneration.

The role of the built environment in causing or exacerbating ill health, environmental degradation, and widening inequalities is well-documented. Attempts to estimate the precise contributions of modifiable environmental factors (e.g. housing, air pollution and transport) toward ill health are difficult; however, a WHO report attributed ‘23% of global deaths and 26% of deaths among children’ to these sources (Prüss-Üstün *et al*. 2016, p.viii). The global construction industry grows apace and is the largest consumer of raw materials (Krausmann *et al*. 2017) and emits roughly 40% of energy-related carbon dioxide emissions, the primary greenhouse gas from human activities (UN Environment and International Energy Agency 2017). Costello *et al*. (2009) called climate change ‘the biggest global health threat of the 21^st^ century’, creating health impacts through ‘changing patterns of disease, water and food insecurity, vulnerable shelter and human settlements, extreme climatic events, and population growth and migration’ (p. 1693). Rapid unplanned urbanisation is implicated in the rise of non-communicable and communicable diseases (Alirol *et al*. 2011, WHO 2014) and increased risk of emerging infectious diseases (Neiderud 2015), such as Covid-19. In addition, the built environment affects health inequities, avoidable differences in health caused by uneven distribution of resources, exemplified by the significant gaps in life expectancy across the least and most deprived in society (CSDH 2008). The built environment contributes to health inequities through a number of pathways including the concentration of environmental burdens (air and noise pollution, limited access to parks, and so on) in low socioeconomic status neighbourhoods (Northridge and Freeman 2011, Gelormino *et al*. 2015). Finally, the design of urban environments can exclude certain groups in society, such as children or people with disabilities, in ways that affect their health and wellbeing (Heylighen *et al*. 2017).

A comprehensive model of interactions across sustainable and healthy urban environment agendas (including how these play out across spatial and temporal scales) is lacking. Such a model is needed to communicate and align action across the disparate professions involved in the built environment sector. Schandl *et al*. (2012) noted a lack of conceptual approaches that cover all relevant urban processes that address human and ecosystem health. The emergence of specific built environment and equity frameworks (Northridge and Freeman 2011, Gelormino *et al*. 2015) demonstrates progress, yet further underscores this sense of fragmentation across the diverse and interconnected factors that affect health in cities. This article and an accompanying paper (Pineo *et al*. 2020a) report on an initiative that aims to fulfil the following objectives: 1) establish how existing healthy urban design and planning frameworks communicate concepts related to scales of health impact and sustainability, equity and inclusion, and their interconnections and 2) develop and test a new framework (the THRIVES Framework) with a range of built environment and health practitioners. The focus of this article is to describe objective one and to introduce the conceptual foundation for the Framework. The THRIVES Framework aims to clarify the following concepts from theory to praxis: the determinants of urban health that can be influenced by urban planning and development; the spatial and temporal scales through which built environment decisions affect health; and the interconnected relations between urban health and sustainability, equity and inclusion. The emphasis is on providing a new tool that reframes existing conceptualisations of healthy urban development, helps stakeholders reach shared understanding of the relevant concepts and guides better policy and design decisions.

This paper builds on theoretical and conceptual approaches from systems thinking, ecological health models and sustainable development. The starting point for this work acknowledges the significant contribution of the Health Map (Barton *et al*. 2003, Barton 2005, Barton and Grant 2006), which itself built on public health models by Hancock (1985) and Dahlgren and Whitehead (1991). This paper describes methods for the production of the THRIVES Framework including a review of contributions from existing frameworks. It introduces field advancements that urge a revision of existing conceptualisations of healthy urban development. The article then describes the THRIVES Framework, detailing the three core principles, three scales of health impact and associated design and planning goals that are achieved across scales of built environment decision-making. Box 1 provides a set of core definitions to ensure the article communicates clearly to diverse research and professional disciplines.

## Methods

Development of the Framework involved four steps: 1) a series of scoping activities including literature review, reflection of the author’s experience and semi-structured interviews 2) a review of existing frameworks 3) identification of Framework components and goals and 4) development and participatory workshop testing with built environment and public health practitioners. This research is informed by theory and concepts from systems thinking (Meadows and Wright 2008), ecological health models (Rayner and Lang 2012), ecosocial epide-miology (Krieger 1994, 2001) and ‘just sustainabilities’ (Agyeman 2013). Descriptions of these, and how they inform the THRIVES Framework, are integrated throughout the paper.

The process of conducting this research is underpinned by action research and transdisciplinary approaches, as described in Pineo *et al*. (2020a). The need for the THRIVES Framework emerged from collaboration between the author and Guy’s and St Thomas’ Charity (GSTC), an urban health charity who want to improve health and wellbeing through their portfolio of land and property. The project involves developing and testing the implementation of this Framework with GSTC and their development partners. The action research and transdisciplinary approach involves the author working across academic disciplines and with non-academic partners to co-develop knowledge and solutions to solve a problem (Meyer 2000, Hall *et al*. 2012). This paper responds to feedback and understanding gained through the collaboration and a formal participatory workshop (Pineo *et al*. 2020a). The author’s own experience working as an urban planner in public and private sector roles has also influenced the arguments set out in this paper.

The action research activities informed a thorough academic and grey literature search to identify existing frameworks. Searches were conducted in Web of Science Core Collection (15 November 2019) and Advanced Google Search (18 November, 5 December 2019) using search terms related to urban environment, health, framework, design and planning. Frameworks were included for further analysis if they met the following criteria: 1) informs how to design and/or plan healthy places (i.e. frameworks that only assess or evaluate design and planning are excluded), 2) published in English in the last 20 years, 3) relates to more than one environmental factor and 4) includes a visual diagram of the relations among environmental and health factors. In relation to point three, there are relevant frameworks that only focus on one aspect of the built environment. These include Lennon *et al*.’s (2017) urban green space affordances framework for health and wellbeing and Tait *et al*.’s (2016) health and mobility infrastructure framework, which both met the other inclusion criteria.

The environmental factors in the THRIVES Framework (called design and planning goals) and scales of health impact/decision-making were identified using the review of other frameworks and Pineo *et al*.’s (2018a) taxonomy of urban health indicators. The topics shown in Table 1 were found in a review of 8006 urban health indicators, the majority of which were evidence-based, and therefore are likely to represent validated measures of urban environment exposures that impact health and wellbeing. Topics were simplified for the Framework, for example, by combining them under umbrella terms (e.g. ‘services’). There is recognition that the Framework visual includes a set of examples, not a comprehensive set of design and planning goals.

cThe current landscape of frameworks

Several important insights were gained from the analysis of existing frameworks (15 were included, out of 1124 search results) regarding topics covered, target audiences and tools provided (see Table 2). The most striking gap was that only three frameworks substantively covered all three concepts of sustainability, equity and inclusion, and in many cases, these topics were discussed in the narrative rather than visually represented in the framework diagram. Table 2 denotes when a concept was minimally described (labelled ‘M’). For instance, sustainable transport networks were the only reference to sustainability in some documents. Most of the frameworks addressed equity with regard to socioeconomic deprivation (12/15), while roughly half (8/15) substantively discussed inclusion. One strong example where inclusion and equity were addressed was in Edwards and Tsouros (2008) framework which explicitly represents population groups including ‘all residents, children and youth, older people, people with disabilities, people with neighbourhoods with low socioeconomic status, other minority and high-risk groups, employees’ (p. 7). In their discussion of how the framework should be applied in planning processes, they highlight the importance of community participation and ‘paying special attention’ to these population groups (p. 6).

It was notable that publications sought to influence a very broad audience of public and private sector practitioners and community members (Table 2). Primary target audiences were those working in the built environment (planners, urban designers, facilities managers, developers, etc.) and health (public health, health promotion, social planning, etc.). However, those working in the development industry (e.g. developers and surveyors) were rarely target audiences (5/15) despite their important role in delivering healthy places. Unsurprisingly, wider city leaders and residents were recognised as key audiences, reflecting the core healthy governance principle of collaboration across sectors and fields. Such collaboration was widely recommended (12/15), including engaging with community members to gather their knowledge about health and place (12/15). Documents routinely included tools to support practitioners such as indicators, checklists and evidence-based policy and design recommendations. In some cases, authors detailed mechanisms to leverage local policy-making structures to support healthy built environments (7/15).

One of the frameworks identified in the review, the Health Map (Barton *et al*. 2003, Barton 2005, Barton and Grant 2006), was regularly referenced in guidance and policy documents, meriting further attention. When introducing a version of the Health Map, Barton (2005) critiqued the lack of integration across the related disciplines of economics, sociology, ecology, and so on, pro-hibiting a consistent perspective for analysis. Crucially, he argued that ‘planning theory and current practice are largely *health-blind*’ citing political, institutional and professional barriers to the integration of health into policy and development (p. 340). Barton saw systems theory as a potential solution to both challenges, drawing on its principles that interconnected elements in a system are interdependent and governed by their interactions.

Systems theories have seen a resurgence since Barton introduced the Health Map, with new proposed applications in urban planning and public policy (Cairney 2012, Price *et al*. 2015, Chettiparamb 2019). Early urban systems modelling approaches were largely dismissed by planners, and Barton noted these limitations (e.g. Taylor 1998). Critiques of Jay Forrester’s Urban Dynamics model (1969) demonstrate why many planners would not have supported his logic, particularly during the onset of post-positivist planning theory (Allmendinger 2002, Rydin 2007). Jhangiani (1976) critiqued Forrester’s model for relying on a policy assumption that moving low-income people out of a city and demolishing their housing would release land for commercial development, reduce local tax burdens and attract higher-earning residents − resulting in ‘a healthy city à la Forrester’ (p. 43). This dismissal of disadvantaged communities is directly opposed to the shared principles of public health and planning. Barton (2005) summarised three failures of systems approaches: not accounting for people and social issues, not positioning settlements in their ecological context, and not reflecting aspatial factors in cities, such as economics. Forrester’s Urban Dynamics model could be characterised as ‘hard’ quantitative systems modelling. Recent applications of this approach draw more on stakeholder knowledge to solve problems rather than modellers’ assumptions, such as Dianati *et al*.’s (2019) participatory analysis of household air pollution in Nairobi’s slums. The Health Map draws on high-level systems theory principles, those that also underpin qualitative ‘soft’ systems thinking (e.g. Meadows and Wright 2008). There are growing calls to draw upon both ‘hard’ and ‘soft’ systems methods to understand and manage urban health (Rydin *et al*. 2012, Gatzweiler *et al*. 2017, 2018).

Other core theories adopted by Barton (2005) are Kevin Lynch’s (1981) theory of the ecosystem applied to cities and insights from human ecologists. The influence of ecosystem theories is evident in the Health Map diagram with its ‘concentric, sector and multi-nodal’ model of social and environmental factors representing their interrelations in ‘complex dynamics’ through an ‘easily-comprehended and descriptive tool’ (Barton 2005, p. 342). The Health Map follows the human ecologists’ understanding that people depend on a functioning ecosystem, as articulated in Hancock’s (1985) Mandala of Health. This perspective is therefore concerned with the natural environment but also a ‘paradigm shift’ in conceptualising the factors and cause and effect pathways that determine individual health and wellbeing from ‘simplistic, reductionist’ to a ‘complex, holistic, interactive, hierarchic systems view known as an ecological model’ (Hancock 1985, p. 1). In addition to these ecological concepts, Barton (2005) was also concerned that the sustainable development agenda had side-lined social dimensions. The Health Map sought to redress this imbalance through explicit focus on health and wellbeing, including concepts of ‘lifestyle, social capital, equity and access’ although he acknowledged that planning’s role in these ‘remains a contested issue’ (Barton 2005, p. 345). A critical review of the Health Map diagram reveals that social issues of equity and inclusion are not explicit.

In view of the article’s aims, this review of frame-works established several core principles and gaps. First, it is clear that the interconnected concepts of sustainability, equity and inclusion need to be more explicitly defined and integrated into a revised frame-work. Second, a healthy urbanism framework should communicate effectively to a wide range of sectors, particularly the development industry which represents key decision-makers. Finally, several theoretical underpinnings of the Health Map should be retained and updated with recent field advancements in health and the built environment. In particular, a revised framework should de-emphasise ‘lifestyle’ and individual choice and further articulate the complex interactions across multiple spatial and temporal scales. The updates to theory and knowledge that underpin the THRIVES Framework are discussed next.

### Field advancements in health and the built environment

As a society, our urban health challenges, or at least our understanding of them, are manifestly different in 2020 compared to the turn of the century, when the Health Map was produced. Notably, media attention to the environment and health surged after the onset of the Covid-19 pandemic with coverage including health inequities (Wall 2020, Singh 2020); the origins of planning, public health and sanitary infrastructure (Klein 2020); and links between the coronavirus and climate crises (Nienaber and Wacket 2020). Putting the global pandemic to one side, other advancements in theory, empirical knowledge, public awareness and wider societal changes prompted the author to develop a revised healthy development framework. Three broad areas of new knowledge and theory require greater representation: 1) the structural factors that determine individual health as opposed to individual characteristics and ‘lifestyle’ choices, 2) the increased urgency of the climate crisis and other environmental breakdown, and 3) the detrimental health impacts of poorly regulated development, particularly for under-represented groups.

### Structural barriers to health

Recent debates in public health and epidemiology argue that ‘lifestyle choices’ are no longer a valid way to conceptualise health risk factors. Individuals may not choose to be inactive or maintain an unhealthy diet, but societal structures dictate these circumstances. The ecological models in Hancock (1985), Dahlgren and Whitehead (1991) and Barton and Grant (2006) put significant attention on societal structures, rejecting the reductionist biomedical model that focuses on the role of individual genetic characteristics in determining health. Despite influential critiques (Engel 1977) and advancements of other perspectives (Lang and Rayner 2012) the biomedical perspective persists today (Farre and Rapley 2017). So too does the notion of unhealthy ‘lifestyles’, when factors of equity and inclusion should be more prominent. Nancy Krieger’s (1994, 2001) ecosocial theory highlights the layering of disadvantage that can affect health throughout a person’s life, from exposure to pollutants during pregnancy through to poverty and race discrimination.

Krieger confronted the gaps in dominant social epidemiology theories by asking about ill health: ‘do the causes lie in bad genes?, bad behaviours? Or accumulations of bad living and working conditions born of egregious social policies, past and present?’ (Krieger 2001, p. 668). Her ecosocial theory would point to an amalgam of these explanations through its constructs of embodiment, cumulative interplay, accountability and agency. Krieger combines biological, ecological and social analyses to explain the determinants of health, arguing ‘we literally incorporate, biologically, the world around us, a world in which we simultaneously are but one biological species among many − and one whose labour and ideas literally have transformed the face of this earth’ (p. 668). The ecological concepts that influenced ecosocial theory, and contemporary ‘eco-epidemiology’ (Susser and Susser 1996) and ‘social-ecological systems perspective’ (McMichael 1999) theories include five core concepts: scale, level of organisation, dynamic states, mathematical modelling and understanding unique phenomena in relation to general processes (Krieger 2001, p. 672). Building upon Barton’s (2005) insights from Lynch and the human ecologists, Krieger’s theory brings additional explanatory power for the complex inter-plays between people and the environment, particularly highlighting structural barriers to health.

The holistic thinking in Krieger’s theory has been matched by new definitions of health and new requirements for public policies. Bircher and Kuruvilla’s (2014) Meikirch Model of Health (see Box 1) emphasises the ‘conducive interactions between individuals’ potentials, life’s demands, and social and environmental determinants’ (p. 363). In line with this argument, Kelly and Russo (2018) use social practice theory to challenge the common public policy narrative that non-communicable diseases will be solved through individual behaviour interventions. Instead, they promote consideration of the wider structural factors influencing drinking, smoking, physical inactivity and healthy eating, including those related to the built environment such as promotion of active travel. Their position is to improve policy responses in line with an ecological health model and to push policymakers beyond rhetoric toward action that involves confronting the vested and powerful interests that determine the main risk factors of chronic diseases and inequalities.

These new perspectives throw into question ecological health models’ central positioning of individuals (and their genetic and constitutional factors) in diagrams of the determinants of health. Putting people at the centre of a model about health is logical, yet it necessarily focuses attention on individual factors as the principal determinant of health. It is argued here that built environment professionals require greater awareness of individuals’ position within society and the natural environment. The language of ‘lifestyles’ found in healthy planning and design guidance is outdated and requires reframing. Urban development stakeholders need to aim higher to remedy unfair differences in access to health-promoting resources at multiple scales, including accessible transport, clean air and quality housing to name a few.

### Urgency of environmental breakdown

The second area of new knowledge and theory that should underpin a revised framework for built environment professionals relates to the significant health impacts of environmental breakdown. Research on the human health impacts of environmental degradation started in the mid-19^th^ century and now devotes significant focus to the climate crisis and other environmental breakdown (Buse *et al*. 2018). The effects of climate change are not only a problem for the future but they are also already happening and they threaten half a century of global development and health gains (Watts *et al*. 2015). This empirical evidence may only go so far in shifting public opinion (and subsequent policy action) about the causes and risks of environmental change, which is significantly influenced by wider factors including beliefs, attitudes, ideology, personal experience and other factors (Howe *et al*. 2015).

Public perception of climate change risks is rapidly changing, highlighting the increased urgency and support for policy action. The majority of respondents to an international YouGov survey in 28 countries believed that climate change would likely lead to serious economic damage, the loss of cities due to sea-level rise, and mass migration; and Asian and Pacific country respondents felt it could likely lead to extinction of the human race (Smith 2019). Even in the USA, which previously lagged behind in public acceptance of anthropogenic climate change, there is majority support for mitigation policies, such as renewable energy (Howe *et al*. 2015). The extensive bushfires across Australia in the summer months spanning 2019 and early 2020 caused a national public reflection on the extreme impacts of the climate crisis and associated government action (Anon 2020). The combination of growing scientific and public understanding of climate change’s health impacts *for current and future populations* demands a stronger reflection of climate science in built environment health guidance. In other words, decisions taken about the built environment will affect human health for local and distant populations over time as a result of the distributed impacts of the climate emergency.

### Urban developments’ impact on under-represented groups

A final area of field advancements relates to increasing knowledge about the health impacts of different forms of urban development and inequitable impacts across specific groups in society. There is increased recognition that market-driven and poorly regulated development can have detrimental health effects, and yet there has been an over-reliance on this form of growth in many countries to meet housing demand and fund urban improvements. Rydin (2013) describes a shift in British planning beginning in the 1970s where reductions in public sector funding were accompanied by a de-valuing of planning’s regulatory role in protecting the public interest. Although these trends play out differently across planning systems, they have generally reduced planners’ ability to leverage positive health outcomes from development (Carmichael *et al*. 2019, Kent and Thompson 2019, Pineo *et al*. 2020b). An adversarial relationship has been documented between planners and developers in negotiations about how urban development will support local health. For example, an Australian planner working near Melbourne described being ‘beholden’ to developers to increase the local housing supply. The planner recalled a case where they requested improvements to a development, including pedestrian-friendly design, and the developer threatened to abandon the project entirely (Pineo *et al*. 2020b, p. 6). Gaps in public sector funding to improve ageing infrastructure or meet local housing demand may be filled by the private sector where a commercial return can be gained, but a growing body of literature raises concerns about the health impacts of these new developments or regenerated areas (Anguelovski *et al*. 2019, Schnake-Mahl *et al*. 2020). These challenges relate to well-known dynamics of power, inequity and the under-representation of groups who are most affected by urban development (Corburn 2013, Bhatia 2014). Although these issues have been simmering for decades, the epidemiological evidence base has recently grown stronger and given weight to the measurable health impacts of such developments.

On top of growing health-related evidence, other societal shifts have pushed new aspects of equity and inclusion to the forefront of healthy urbanism debates. Scholars and practitioners are increasingly exposing the unfair (health) impacts of urban development caused by failure to design for residents’ diverse needs with regard to gender, age, race and other characteristics. Criado-Perez’s (2019) book exposes the impact felt by women when built environment design and management strategies do not account for their needs. For example, she highlights Loukaitou-Sideris and Fink’s (2008) research on the mismatch between women’s safety needs on public transit and the strategies adopted by transit operators. The implications of this mismatch are clearly linked to women’s physical and mental health − if they are deterred from transport they may be less active (and therefore at higher risk for chronic diseases) and have reduced access to employment opportunities. Similarly, Lusk *et al*. (2019) documented the bicycle infrastructure needs of low-income minority communities in Boston and found their requirements to be absent from transport and Crime Prevention Through Environmental Design (CPTED) guidelines, representing the likely ubiquity of exclusive mobility infrastructure. Beyond a narrow focus on disabilities, recognition that some groups in society need different design features to live a healthy life is a recent shift in built environment theory and practice.

The proposed THRIVES Framework responds to the advancements in theory, science and practice outlined above and the previously described gaps in existing frameworks. In the author’s view, there is an urgent need to reframe health and its wider determinants for built environment audiences. There is a need to more fully integrate the complex interconnections across sustainability, equity and inclusion concepts to demonstrate how urban policy and development affect health locally and remotely in temporal and spatial terms. The next section introduces the THRIVES Framework with and explanation of its component parts in terms of theory and practical implications.

### The THRIVES Framework

The THRIVES (Towards Healthy uRbanism: InclusiVe Equitable Sustainable) Framework offers a new way of conceptualising the interconnected health impacts of built environment policies and design decisions at multiple urban scales. The Framework can be used to inform research and practice in the fields of urban planning, architecture, urban design, engineering, transport, public health and others (including all of the actors in Table 2).

**Figure 1:**
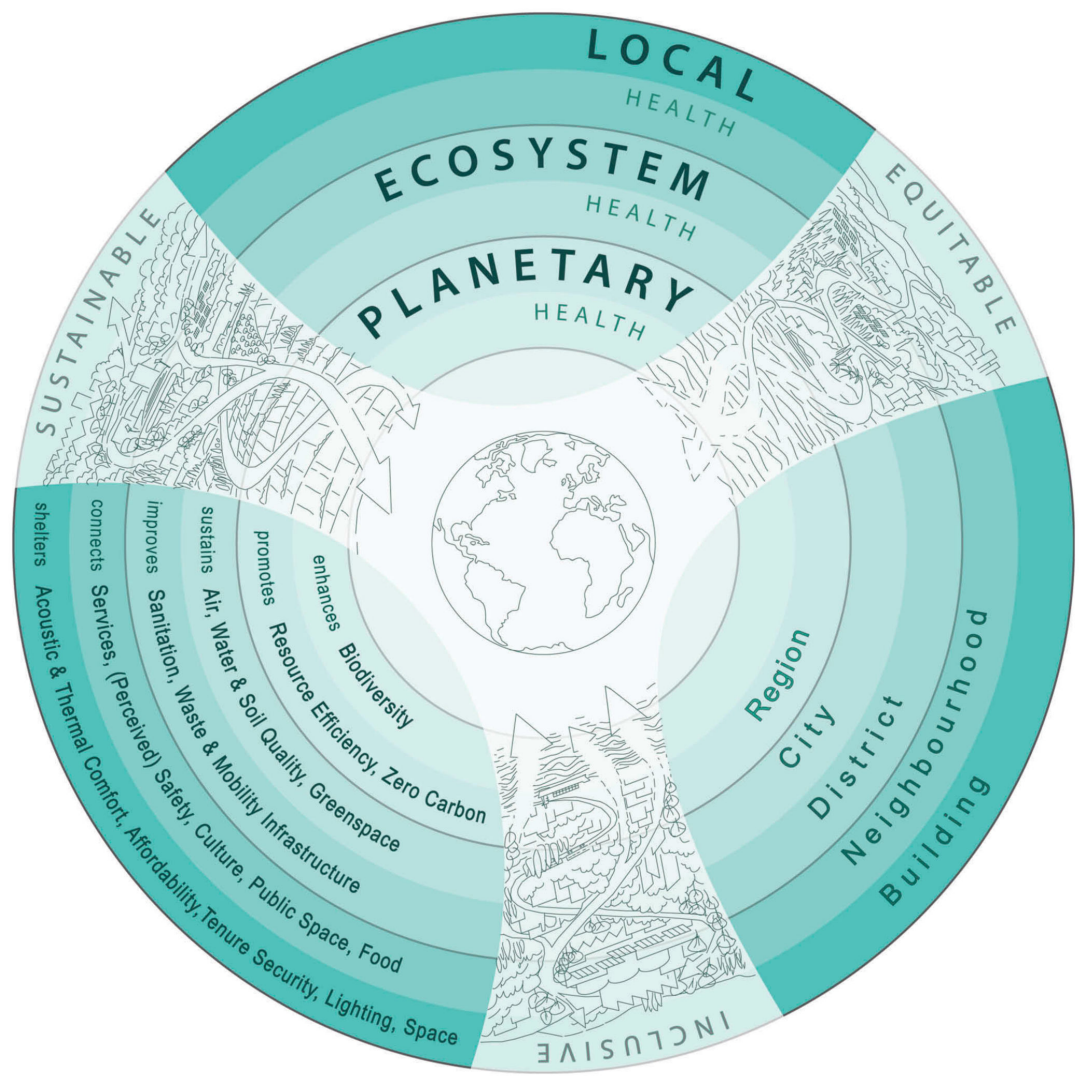
THRIVES Framework (Towards Healthy uRbanism: InclusiVe Equitable Sustainable).

The visual representation of the Framework (Figure 1) uses words and images to evoke processes and outcomes of healthy urbanism. The visual illustrations show the planet at the centre which is connected to regional, periurban and urban landscapes moving outward. The illustrated spatial scales align to the three scales of health impact (planetary, ecosystem and local). Concentric rings in the image demonstrate interconnections across each scale and help to visually align the scales of health impact with scales of urban decision-making related to the built environment. The latter begin with regional (in recognition of cities’ role in their wider geographical region) and then move to city, district, neighbourhood and building scales. Decision-making scales will vary across cities and forms of development. The Frameworks seeks to represent scales that are indicative of policy or decision-making for typical government tiers or types of urban development. The scales of health impact and decision-making are associated with evidence-based design and planning goals. All decision-making should be informed by three core principles that define what healthy urbanism ‘looks like’: inclusive, equitable and sustainable.

The Framework aims to communicate three broad propositions. First, there are complex interactions between urban environments and health that result in health impacts at multiple spatial and temporal scales that may not be immediately obvious to designers and policymakers. Interactions across scales are visually represented in THRIVES through the nested circles and the arrows intersecting the illustrations. Pineo *et al*. (2018b) summarise key characteristics of complex systems as they relate to healthy urbanism. Complex systems are dynamic, comprised of many elements, interconnected and governed by feedback. Such systems contain non-linear structure and result in emergent behaviour and counterintuitive results. Achieving healthy urbanism requires systems thinking approaches, integrated design and interdisciplinary working. Applying a systems approach will result in many co-benefits (or win-win-win solutions) for social, economic and environmental goals. Second, knowledge to inform decisions about health and urban environments should come from multiple sources, including scientific (and other technical) evidence and locally affected communities. This will ensure that urban development better serves groups which have historically been under-represented in design, such as women, minorities and children. Finally, urban environments and their associated health and wellbeing impacts should be monitored by government, building managers and other responsible authorities (again using participatory processes to define indicators) to ensure that policy and design intentions result in health-promoting places for all residents. The following sections describe the Framework, explaining how urban policy and design decisions will affect peoples’ health and well-being through diverse mechanisms, resulting in impacts that may be spatially or temporally distant to where decisions are made.

### Three core principles

To the extent that it is possible, all design and policy decisions should be inclusive, equitable and sustainable. In the author’s experience, these three concepts are viewed as independent goals by built environment professionals and they are often the remit of specialist consultants. A holistic understanding of the interconnections between these principles is lacking, resulting in a narrow view of how the urban environment affects health. This section makes the argument that each principle is essential to healthy urbanism by providing definitions, relations to health and underlying theoretical concepts.

### Inclusive

Inclusive urban environments represent both an outcome to strive for and a process to follow (Heylighen *et al*. 2017). Built environment professionals have varied understandings of inclusive design and how it should be applied, leading to limited adoption of this principle to date (Heylighen *et al*. 2017). Furthermore, developers and property owners have been reluctant to respond to statutory accessibility requirements, often citing cost as the principal barrier (Mitchell *et al*. 2003). The topic is historically rooted in designing environments that include people of all physical abilities and ages. Even this narrow definition put significant responsibility on design professionals as Clarkson and Coleman (2010) argued that people are not disabled by ‘physical and mental impediments’ but ‘by designs and environments that do not take account of the full range of human capabilities’ (p. 127). Global trends demand inclusive design as the ageing population and those with chronic conditions are more likely to have physical impairments that require built environment design responses such as sloped level changes and more places to rest in public spaces, among other measures. The concept of inclusion has broadened over time and it now includes factors such as gender and race. Design for all means ‘design for human diversity, social inclusion and equality’ (EIDD 2009). As argued in the previous section, the built environment design needs of women and minorities have been largely ignored, resulting in potential negative health impacts.

In the THRIVES Framework, an inclusive built environment enables all members of society to conveniently participate in daily activities without feeling that they are disadvantaged by their personal characteristics or needs, and this is achieved through participatory processes. This definition builds on existing definitions (EIDD 2009, Heylighen *et al*. 2017) and highlights a subjective element because the emotions associated with feeling excluded are also damaging for health and wellbeing (Marsh *et al*. 2020a). Individual characteristics that require design considerations will vary across projects and internationally. A useful starting point for characteristics to consider are those protected under the UK’s Equality Act 2010: age, disability, gender reassignment, marriage and civil partnership, pregnancy and maternity, race, religion or belief, sex and sexual orientation. In addition, built environment design and policy should consider the requirements of people with: physical and mental health conditions that are not classified as disabilities, such as declining cognitive function associated with dementia; people with several of these characteristics who may feel particularly excluded; and other vulnerable people such as homeless or low-income communities.

Healthy urbanism requires the participation of all members of society in design and planning processes. Kumar (2002) claimed ‘that development cannot be sustainable and long-lasting unless people’s participation is made central to the development process’ (p. 23). The need to integrate community knowledge in design and planning to promote health was articulated through the transport examples from Loukaitou-Sideris and Fink’s (2008) research on women and public transit and Lusk *et al*.’s (2019) work on the cycling infrastructure needs of low-income minority communities. Actively seeking community knowledge about health and place is important because their perspectives may differ to those of design professionals (Dennis *et al*. 2009). Inclusive urban development processes build on the participation, co-production and co-design principles of health (Martin 2009, Israel *et al*. 2019) and urban design and planning (Kumar 2002, Innes and Booher 2010, Agyeman 2013). Groups that will be affected by urban policies and designs should participate in deliberations over what is proposed and how it will be achieved. Furthermore, citizen groups may take the lead through various self-build and community-led development approaches (Agyeman 2013).

### Equitable

The broad concept of equity is about fair distribution of resources, but THRIVES focuses on health equity, or the absence of unfair differences in health that are caused by uneven distribution of health-promoting resources in society (WHO 2020). Braveman (2014) argues that achieving health equity requires ‘striving for the highest possible standard of health for all people and giving special attention to the needs of those at greatest risk of poor health, based on social conditions’ (p. 6). Income inequalities reduce population health and wellbeing (Pickett and Wilkinson 2015) and people living in poorer neighbourhoods tend to die earlier and spend more time in ill health than people living in more affluent neighbourhoods − known as the social gradient in health (CSDH 2008, Marmot *et al*. 2010, 2020). The relations between income inequalities and health are not part of core educational curricula for most built environment professionals. Therefore, these practitioners are also unlikely to draw connections between poverty and other social and environmental inequalities that influence health and wellbeing in cities, such as reduced access to healthy housing, green infrastructure and employment (Bambra *et al*. 2010, Geddes *et al*. 2011, WHO 2012).

There is a strong evidence base outlining the health impacts of reduced access to health-promoting environmental resources. Residents in low-income or ethnic minority neighbourhoods have reduced access to healthy foods and greater access to unhealthy foods (Fraser *et al*. 2010, Black *et al*. 2014). Low-income communities are more likely than affluent communities to have increased exposure to air pollution (WHO 2013), noise pollution (Nega *et al*. 2013) and rates of road traffic injuries (Cairns *et al*. 2015). They are more likely to live in poor quality housing (Braubach *et al*. 2011) that is overcrowded and less able to withstand extreme heat, cold, flooding, earth-quakes and natural disasters (WHO 2016, 2018b). Deprived communities also have reduced access to green space (Institute of Health Equity 2014), fewer recreation facilities (Gordon-Larsen *et al*. 2006, Hillsdon *et al*. 2007), and reduced availability of children’s play areas (Curtice *et al*. 2005). All of these factors are directly influenced by built environment decisions and they should therefore be a core consideration in urban planning and development.

In the THRIVES Framework, an equitable environment gives access to health-promoting environments to all residents and specifically considers and seeks to reduce barriers to access (be they physical, cultural, social or economic). The public health principle of ‘proportionate universalism’ offers an approach to reduce inequity that can be applied to urban policy and development (Carey *et al*. 2015, Egan *et al*. 2016). Adopting Carey et al.’s approach to proportionate universalism for healthy urban environments would involve measures that are universal (applied to every-body in society, such as safe water) and targeted (applied to those with the most need, such as highquality social housing). It would also mean adopting the governance principle of ‘subsidiarity’, where residents are closely involved in determining solutions to the challenges they face (as argued under the inclusion principle above). There are overlaps between the concepts of inclusion and equity. For the purposes of the THRIVES Framework, equity is about overcoming unfair distribution of resources, whilst inclusion is about ensuring everybody can fully participate in society and daily activities, achieved through a process of participatory design and planning.

### Sustainable

The final core principle in the THRIVES Framework is sustainability. The term sustainability and the commonly referenced ‘triple bottom line’ balancing of economic, environmental and social objectives are contested and complex (Joss 2015). Raworth (2017) argues that continued economic development is not compatible with the limited resources of the planet. However, it may still be required in many parts of the world, particularly low-income settings that rely on development to reduce poverty and improve quality of life (Agyeman 2013). High-income countries, such as the UK and Australia, have also relied on growth to fund community benefits through new development and the success and long-term viability of this has been called into question (Rydin 2013). The UN Sustainable Development Goals (SDGs) (United Nations General Assembly 2015) provide a foundation for combining urban health and sustainability agendas from global to local scales. The SDGs attribute greater importance to both environmental limits and the role of urban policy than previous global sustainable development initiatives (Parnell 2016) and they are aligned to health equity (Marmot and Bell 2018).

The THRIVES Framework considers sustainable development to be supportive of the needs of the current (and immediately local) population without compromising the needs of future (or spatially distant) populations, building on the Brundtland Report definition (World Commission on Environment and Development 1987). The Framework builds upon Agyeman’s (2013) concept of ‘just sustainabilities’, simplified to ‘just sustainability’ by Rydin (2013). This broad view of sustainability is about process and outcome, and it relates to improving wellbeing, equity and justice for current and future generations, and doing so within ecosystem limits. Agyeman (2013) emphasises that these principles are mutually supportive, stating that ‘both social and environmental health are dependent, to a large extent, on greater justice and equality’ (p. 18). The principles of just sustainabilities are articulated through both the three core principles and the three scales of health impact in the THRIVES Framework.

### Three scales of health impact

There are three interconnected scales of health impact: planetary health, ecosystem health and local health (Figure 1). In contrast to other frameworks that start with individual characteristics, THRIVES positions core principles and planetary health at its core, shifting the focus from individuals to our environment. Impacts across these scales are not all immediate and may occur over months, years or decades, within and beyond the boundaries of physical environment changes. Built environment decisions often result in changes that last decades or longer, and it is therefore essential to consider how they will impact health at multiple spatial and temporal scales. The author argues that measuring the health impact of urban policy and development (e.g. through health impact assessment or healthy building rating tools) is typically limited to consideration of effects that are spatially and temporally proximate to the proposed change. To create healthy places, professionals must consider impacts at broader scales. It is not necessary to model such effects for all projects − researchers have already created the evidence base that can inform policy and design decisions. This evidence is described below and grouped into three broad scales that create a design heuristic to inform practice.

### Planetary health

The concept of planetary health refers to ‘the health of human civilisation and the state of the natural systems on which it depends’ (Whitmee *et al*. 2015, p. 1974). The built environment causes environmental degradation at a global scale through resource-intensive design, construction and operation (Whitmee *et al*. 2015) and through habitat destruction that reduces biodiversity (Opoku 2019). Unplanned rapid urbanisation and deforestation also results in people living closer to ‘untouched ecosystems’ where there is greater risk for transfer of zoonotic diseases (Neiderud 2015, p. 6). Local and global environmental harms affect human health in multiple interrelated ways and the impacts can be felt in distant locations to such harms (Díaz *et al*. 2015). Environmental pollution has often affected poor populations disproportionately to richer groups and this inequity plays out at multiple scales within cities and internationally. Low-income groups have contributed the least carbon emissions (and other harms) but will suffer the most from the health impacts of climate change, such as malnutrition and death or injury from extreme weather (Butler 2016).

The built environment can support planetary health through three goals (intended as key examples): *enhancing* biodiversity and *promoting* resource efficiency and zero carbon (Figure 1). At the urban scale, the most significant design and planning decisions that affect planetary health are made at the regional to city policy levels. Younger *et al*. (2008) describe how health is supported by reducing carbon emissions through land use and transport planning. Reducing motor vehicle travel supports climate change mitigation, reduces injuries and supports active travel. In turn, reducing traffic pollution leads to reduced noncommunicable diseases and increased social capital which improves mental health and wellbeing. Decreasing traffic will also support more inclusive cities, recognising that road injuries are the biggest cause of death in people aged 5 to 29 (WHO 2018a). Other scales of urban decision-making are relevant to support planetary health (all of the scales are interconnected). For example, striving for a zero carbon built environment would mean increasing energy efficiency in buildings which can improve thermal comfort, thus demonstrating the interconnections across multiple health impact scales.

### Ecosystem health

Ecosystem health as a concept relates more to the health of the environment than the connection between the environment and human health; however, the THRIVES Framework emphasises the latter. Ecosystems are ‘webs of connections between living and non-living system components’ and they are foundational to human health (Buse *et al*. 2018). As with planetary health, there is a complex system of interconnections between ecosystem and human health. Under an ecosystem services approach, ecosystems provide food, clean water, climate regulation and recreational opportunities, and in turn, these services support human health and wellbeing (Haines-Young and Potschin 2010). Similarly to planetary health, harms to ecosystem health are felt most closely by low-income populations who depend heavily on their local environment, such as subsistence farmers (Butler 2016).

The THRIVES Framework lists five example goals for ecosystem health: *sustaining* air, water and soil quality, and greenspace, and *improving* sanitation, waste, and mobility infrastructure. Highly impactful decisions that affect ecosystem health are made at the scales of regional to district policy and design − a point illustrated through the design and planning goal of water quality. Water is a necessity for human and ecosystem health, and urban growth increases water demand (McDonald *et al*. 2014), which will be exacerbated by climate change (Schultz 2019). One in four global cities is water stressed, increasing the importance of sustainable water management (McDonald *et al*. 2014), such as through avoiding development in flood-prone areas, using permeable landscape surfaces, green roofs and other landscaping to slow down water drainage and installing rainwater harvesting systems to collect and use water (Lamond 2015). Improving sanitation and waste infrastructure will protect water quality and reduce exposure to infectious diseases (Alirol *et al*. 2011).

### Local health

The final scale of health impact is local, and it contains two rings in Figure 1, representing design and planning decisions for buildings and neighbourhoods (the outermost ring and penultimate ring, respectively). At the neighbourhood scale, example design and planning goals are to *connect* people with services (covering employment, education, retail, leisure, healthcare and other facilities), perceived and actual safety, culture, public space and food. At the building scale, example goals are to *shelter* people with acoustic and thermal comfort, affordability, tenure security, lighting and space. There are many pathways through which different types of buildings (e.g. offices, homes, schools, hospitals, etc.) and neighbourhood design (e.g. compact or sprawling urban form) influence health. A large body of literature (see Rogerson *et al*. 2014, Bird *et al*. 2018) and local knowledge (from professionals and community groups) can be used to further prioritise or complement the example design and planning goals in THRIVES.

As with planetary and ecosystem health, this scale interacts heavily across the others. A healthy building or neighbourhood must support human health alongside ecosystem and planetary health, as argued by Levin (1995). For instance, creating walkable neighbourhood environments will support physical activity and social connection whilst reducing local air pollution and greenhouse gas emissions. Walkability is achieved through decisions taken at multiple scales including urban land-use policies, mobility infrastructure, location of services and quality of public space. In most cases, the author argues that health and sustainability are mutually reinforcing design and planning goals, however, there may be some tensions that bare consideration. At the building scale, increased energy efficiency could result in overheating or poor air quality (Shrubsole *et al*. 2014), conversely efforts to filter out air pollutants could increase energy use in buildings. Integrated design and planning should be adopted at all scales to avoid these tensions wherever possible. The next section highlights the potential co-benefits of healthy urbanism by examining two urban development scenarios at neighbourhood and building scales (Table 3) through the lens of the THRIVES Framework.

### Moving from theory to praxis

A primary goal of the THRIVES Framework is to reframe current perspectives on how the built environment affects health and wellbeing, ultimately shifting practice in urban planning, design and development. Several hypothetical development scenarios are used here to illustrate practical application of the Framework. A brief summary of the participatory workshop feedback follows, outlining potential paths to implementation.

The process of moving from the conceptual framing of the THRIVES Framework to actual policy and design decisions is explained through two hypothetical urban development scenarios of different scales and types (Table 3). Each scenario is explored through a single design strategy that produces health benefits across the three core principles and scales of health impact. These benefits are not achieved automatically and the table is not comprehensive in assessing health impacts. Design strategies need to respond to the local context and be informed by community knowledge. In the first scenario, a local authority has received funding to improve mobility infrastructure in a deprived neighbourhood. They achieve this goal by installing new solar-powered street lighting, pedestrian crossings, cycle lanes and street furniture (such as benches and planters). In the second scenario, a commercial developer has partnered with a local authority to refurbish and expand a 1960s housing estate with a mixture of market-rate and affordable properties. Their approach involves upgrading energy efficiency and ventilation in dwellings, providing publicly accessible outdoor play space, and installing new sustainable drainage and ground-floor storage for cycles and strollers. All of these measures will support health, but Table 3 shows how viewing a single strategy for each project through the THRIVES lens uncovers a much broader range of health and wellbeing impacts than would typically be considered in such projects.

The worked examples in Table 3 show several insights that can emerge from considering urban development through the THRIVES Framework. First, applying a systems thinking lens shows that even relatively minor changes to the urban environment can support health. The solar-powered street lights (scenario one) would typically be considered as a strategy to save money and reduce carbon emissions by a city’s transport department, yet there are many potential health benefits. An inclusive process, such as a community street audit, can uncover current problems related to safety concerns, the placement of lights, crossings and street furniture. Systems thinking principles highlight that the new lighting may be necessary, but not sufficient, to bring about greater physical activity. Activity can be seen as an emergent behaviour resulting from an interconnected mobility system (Pineo *et al*. 2018b), in which street lighting is one important element. Second, it is apparent that each design strategy can affect human health at multiple spatial and temporal scales. Scenario two describes planetary, ecosystem and local health impacts arising from a new play space, many of which are unlikely to be considered in the typical design process. By considering such impacts, design teams can specify appropriate materials (e.g. permeable surfaces) and landscaping (e.g. to provide shading). Third, examining design strategies through the THRIVES lens may highlight conflicts between goals or the need for compromises. An inclusive process can help design teams identify or prioritise suitable solutions in the context of limited budgets or other constraints. In both scenarios, monitoring can ensure that design intentions have been achieved over time.

Professionals who attended the participatory work-shop gave several ideas for how the THRIVES Framework could be used to inform practice (Pineo *et al*. 2020a). The participants included a mix of built environment and public health professionals working in the public and private sector in England. Participants identified the Framework’s potential impact in changing professionals’ perspectives on how urban development impacts health, expanding existing conceptualisations to a broader and more holistic set of topics. Professionals thought the Framework could break down disciplinary barriers and help to build a shared understanding of healthy urbanism. Finally, they listed multiple practical uses of THRIVES, including informing local planning policy, supporting impact assessments (health, environmental, integrated, etc.), and aligning development proposals to specific health goals. There was critical feedback about the preliminary version of the Framework that was used to iterate and improve the version presented in this article, see Pineo *et al*. (2020a). A significant focus of criticism related to a desire for monitoring indicators, design checklists and other guidance to apply the conceptual tool in practice.

Moving from theory to practice with the THRIVES Framework will require more than the publication of a high-level conceptual diagram. An advisory group has been convened to guide the co-development of a short training programme with relevant professionals.^1^ The roles of different actors will vary when using THRIVES and this requires further exploration in terms of their diverse perceptions and requirements. To that end, the THRIVES Framework is being tested through implementation in the design and planning process of a large-scale urban development in London (see Methods). It is hoped that the training programme and additional insights gained through using THRIVES will lead to greater understanding of the mechanisms that are successful at improving health through urban development over time. In this way, the author proposes that the Framework may inform research about the process and outcomes of healthy urban development.

## Conclusion

The predicted growth in urban areas demonstrates the significant opportunity that built environment professionals have to shape future physical and natural infrastructure in a way that will positively impact people and the planet. The THRIVES Framework can support this task. As Kate Raworth (2017) claims ‘human thriving depends upon planetary thriving’, yet not all in society agree to work within the planetary and human wellbeing boundaries articulated in her doughnut framework (p. 50). In response to this, THRIVES could be useful to support interdisciplinary collaboration and build shared understanding among practitioners working on design, policy development or impact assessment (Pineo *et al*. 2020a). Recommendations for professionals arising from this research are to use THRIVES as the basis for discussions with partners or within teams. These conversations could begin with participants sharing their views about how their work affects the different scales of health impact and the three core principles. The Framework could also be used to conduct a mapping exercise of how design or policy strategies affect health (as in Table 3). These activities may reveal gaps, but they may also show that professionals were unknowingly doing work that supports health.

In this paper, the author has argued for a reframing of healthy urbanism to inform built environment research and practice. The proposed THRIVES Framework fills gaps in previous conceptualisations and responds to field advancements across diverse sectors in science and society. The novel contributions of THRIVES are its assemblage and visual portrayal of diverse theory and empirical evidence about health and the urban environment, aimed at (and tested with) a built environment audience, specifically the following three insights. First, THRIVES highlights the structural factors that determine health and de-emphasises individual ‘lifestyle’ choices. Second, it proposes greater attention to the complex links between local, ecosystem and planetary health that result in health effects that are both proximate and distant in space and time to urban development. Third, it explicates the interconnected principles of equity, inclusion and sustainability that are not typically understood as key requirements for health. The concepts described in this paper are too often siloed, diluting the arguments and resources to build and maintain healthy places. The THRIVES Framework offers a holistic conceptualisation of healthy urban environments that can build shared understanding among researchers and practitioners.

## Figures and Tables

**Table 1:** List of topics measured in urban health indicators within four sub-classes of Pineo et al.’s (2018a) taxonomy.

Scope sub-classes	Topics
Environment	Transport, housing, water quality, air quality, land use, services and utilities, food environment, urban design, natural environment, pollutants, public open space, waste management, and noise
Social	Crime and safety, education, behaviours, leisure and culture, demographics, social networks, local democracy, disasters, and other
Health	Health outcomes, health and social services
Economic	Employment/income, and economy

**Table 2:** Analysis of included frameworks including target audiences, practices they advocate, tools they include, and topics they describe.

Framework publication	Target Audiences										Recommends…					Includes…					Describes…				
	Planning	Development	Other BE	Health General	Public health	City departments	City leaders	Community members	Other	Collaboration	Community knowledge	HIA or other assessment	Monitoring	Indicators	Checklist	EBP & design measures	Case studies	Co-benefits	Policy structures	Equity	Inclusion	Sustainability
Edwards and Tsouros (2008)				Y		Y	Y	Y	Y	Y	Y	Y	Y	Y	Y	Y	Y	Y	Gen	Y	Y	M
Sport England (2015)	Y	Y	Y	Y				Y			Y	M	Y	Y	N	Y	Y	Y	Y	CS	Y	Y	M
Pineo *et al.* (2018b)			Y				Y				Y	N	N	Y		Y	N	N	M		Y	N	Y	N	M
BC Centre for Disease Control (2018)	Y	Y		Y		Y		Y			N	N	N	N		N	N	Y	M		Y	CS	Y	Y	M
National Heart Foundation of Australia (Victorian Division) (2012)	Y		Y	Y	Y	Y					Y	Y	Y	N		N	Y	Y	Y		Y	CS	N	M	M
CIP and HP Lanare-Golders (n.d.)	Y			Y				Y	Y		Y	Y	Y	Y		Y	Y	Y	Y		Y	CS	Y	M	Y
Gehl Institute (2018)	Y		Y		Y		Y	Y			Y	Y	N	Y		Y	N	Y	Y		N	N	Y	Y	N
Northridge *et al.* (2003); Northridge and Sclar (2003)	Y				Y						Y	Y	Y	Y		Y	N	N	N		Y	N	Y	N	Y
Mithun Inc. and Denver Housing Authority (2012)									Y		Y	Y	Y	Y		Y	N	Y	Y		N	CS	Y	Y	Y
Ross and Chang (2014)	Y		Y		Y		Y	Y			Y	Y	Y	Y		N	Y	Y	Y		M	CS	Y	M	M
TCPA (2017)	Y	Y	Y	Y	Y						Y	M	Y	Y		N	Y	Y	Y		Y	CS	Y	Y	Y
Barton (2005; Barton and Grant (2006)	Y		Y		Y	Y			Y		Y	Y	Y	Y		N	N	N	N		Y	N	Y	Y	Y
Pineo and Rydin (2018)		Y	Y								N	Y	Y	Y		N	N	Y	Y		Y	N	Y	N	Y
Rajan and Manoj Kumar (2016)			Y								N	N	N	N		N	N	Y	N		N	N	N	N	M
UK Green Building Council (2016)		Y	Y								Y	Y	Y	Y		Y	N	Y	Y		Y	N	N	Y	Y

BE: built environment, M: minimally, EBP: evidence-based policy, Gen: general, CS: context specific, Y: yes, N: no.

**Table 3:** Evaluation of two design strategies using the THRIVES Framework scales of health impact and core principles.

Framework principles and scales of health impact		Example urban development scenarios, selected design strategies and associated health fits		
		Scenario 1: Improvements to neighbourhood-scale mobility infrastructure.	Scenario 2: Redevelopment of a 1960s housing estate.
		Design strategy: New solar-powered street lighting	Design strategy: Publicly accessible outhoor play space
Core principles	Inclusive − Process	Community street audits can identify where to put lights to best support local needs.	Co-design process can inform factors such as prioritisation of play space features, including target ages.
	Inclusive − Outcome	Improved sense of safety (from more street lighting) leads to increased physical activity and social interaction for all residents, including disabled, women, elderly, and children.	Installation of features such as benches, shading, water fountain (etc.) supports the needs of vulnerable residents, including children, grandparents and nursing mothers.
	Equitable	In addition to the above, increased sense of safety in deprived community leads to opportunities (e.g. jobs, schools, etc.) in wider areas.	Creation of publicly accessible on-site amenities in the play space benefits low- income residents, within and beyond the estate.
	Sustainable	Reduced fossil fuel use (to power lighting) lowers pollution, while supporting a green lighting business and associated jobs.	Reduced flooding and car use (see below) leads to reductions in air/water pollution and carbon emissions.
Scales of health impact	Planetary health	Reduced fossil fuel use leads to lower pollution, thereby decreasing health impacts of climate change.	Local amenity reduces the need to drive to parks, thereby reducing health impacts caused by climate change and air pollution. Sourcing sustainable/ethical materials reduces pollution and negative impacts in the supply chain.
	Ecosystem health	Reduced pollution (from switching to renewable energy) increases health benefits from functioning ecosystem.	Permeable surfaces and planting in the play space can be part of sustainable drainage systems, reducing flooding and water pollution, thereby leading to ecosystem health benefits.
	Local health − neighbourhood scale	Improved sense of safety results in greater physical activity and social interaction through incidental interactions.	Play space provides local amenity to increase physical activity and social interaction for children and parents (within and beyond the estate’s boundary).
	Local health − building scale	Lights can be placed/angled to reduce light pollution in buildings.	Green infrastructure in the play space can decrease the urban heat island effect, resulting in better thermal comfort in homes.

## Box 1 Foundation definitions for this article

**Built environment professions** include architects, construction managers, engineers, facilities managers, landscape architects, planners, project managers, surveyors, and urban designers (Construction Industry Council n.d.).

**Framework** is a visual diagram that represents relations among different factors or concepts. It ‘can provide a scaffold and common language to conceptualize the system’ and should provide clear definitions for all components to avoid extended debates during its implementation (Yee *et al*. 2012, p. 415).

**Health and wellbeing** refers to physical and mental health and wellbeing together, recognising their determinants as being a wide set of individual characteristics and societally influenced factors. This builds on the recognised deficiencies of the WHO (1946) definition, instead adopting Bircher and Kuruvilla (2014) definition of health as ‘a state of wellbeing emergent from conducive interactions between individuals’ potentials, life’s demands, and social and environmental determinants’ (p. 363).

**Urban development** is ‘the process of physically producing the built environment, by bringing together multiple actors from construction companies to development financiers to local planners and others’ (Rydin 2010, p. 15). Development encompasses all property sectors and infrastructure and may be large or small in scale, including major urban redevelopment projects and incremental improvements to existing buildings.

## Notes

1 The development of the training programme has been funded by a Bartlett Innovation Fund award at University College London. The advisory group includes representatives from Faculty of Public Health, Guy’s and St Thomas’ Charity, London Healthy Place Network, Public Health England, Royal Institution for Chartered Surveyors, Royal Town Planning Institute, Town & Country Planning Association and other organisations.
